# Supplementary material for: Mitigating Age-Related Ovarian Dysfunction with the Anti-Inflammatory Agent MIT-001
Source: Int J Mol Sci. 2023 Oct 13;24(20):15158. doi: 10.3390/ijms242015158 (PMC10607328; doi:10.3390/ijms242015158)
Supplement: Supplementary file 1 [file ijms-24-15158-s001.zip › supplement Table S2 JHL.pdf]

Supplement table 1

Primer	Gene		Sequence (5'-3')
**β-actin**	beta-actin	F	CAT TGC TGA CAG GAT GCA GAA GG
		R	TGC TGG AAG GTG GAC AGT GAG G
**Ptpn6**	Protein Tyrosine Phosphatase Non-Receptor Type 6	F	GGA CTT CTA TGA CCT GTA CGG A
		R	CGA GCA GTT CAG TGG GTA CTT
**Fcer1g**	Fc Epsilon Receptor 1g	F	ATC TCA GCC GTG ATC TTG TTC T
		R	ACC ATA CAA AAA CAG GAC AGC AT
**Tyrobp**	Transmembrane Immune Signaling Adaptor TYROBP	F	CCC AAG ATG CGA CTG TTC TTC
		R	GTC CCT TGA CCT CGG GAG A
**Slamf9**	SLAM family member 9	F	AGA AAA GCT ATG AGA CGC C
		R	AGG ACC TCA AAA GCC AAT C
**Clec4a3**	C-type lectin domain family 4, member a3	F	ACT TCA ACT GAC TTG GTG G
		R	AAA TCC TGT TCT TCC TGG C
